# Observations on the Treatment of Lateral Curvature of the Spine, &c.

**Published:** 1847-10

**Authors:** 


### Art. XX.

*Observations on the Treatment of Lateral Curvature of the Spine, &c.; with Woodcuts.* By Edward F. Lonsdale, Fellow of the Royal College of Surgeons, &c. &c.—*London,* 1847. 8vo, pp. 116.

Mr. Lonsdale has added another work to the number already treating of deformities of the vertebral column. Like his predecessors, he has his theories, his mechanical supports, his couch, and his rules for muscular exercise.

His theories are of the mechanical order. The causes of lateral curvature, and of curvature to the right side in especial, are pointed out to be, first, employments requiring constrained positions, and in which the right arm is principally in use, and which leads to imperfect expansion of the left side of the thorax and a pendulous position of the left arm, so that it acts as a dead weight on the vertebral column. The original normal difference in the size of the two lungs may, he thinks, be also stated as a predisposing cause of lateral curvature to the right side.

The constriction of the thorax, by stays, is also less resisted by the ribs of the left side than of the right, in consequence of this inferiority in the expansive capacity of the left lung. The more frequent use of the right side than of the left is another cause, inasmuch as this is followed by a superior development of the muscles of that side, and a freer expansion of the lungs. Then Mr. Lonsdale refers to the greater development of the liver on the right side; this prevents the ribs of that side giving way. The liver, in fact, acting as a mechanical support, a sort of pad to the ribs, and even predisposing to the right-handedness of the majority of people. Mr. Lonsdale boldly denies, however, that there is any evidence or reason to suppose that the muscles on the one side are weaker than on the other.

From these and other remarks, and from certain faults of omission too, we infer that Mr. Lonsdale's physiological knowledge is not profound, and that he has little if any acquaintance with French and German writers on the subject of spinal deformities. In the first place, the inequality in the vital powers of the two symmetrical halves of the body is a demonstrated general fact in physiology. Mr. Lonsdale will find numerous instances illustrative of this fact in physiological works, some of which are stated in Dr. Laycock's 'Treatise on the Nervous Diseases of Women.' In the embryo the liver is a symmetrical organ; it assumes its usual form from shrinking of the left half. In air-breathing gasteropoda and in serpents with one lung only, it is the left side that is defective; the pulmonary apparatus is on the right side, so in other organs, as the testes, mammæ, &c. In females the thoracic development is less energetic than in men, and hence a proclivity to those deformities of the thorax, and displacement of the thoracic vertebræ which are dependent on irregular muscular action. All this seems to be unknown to Mr Lonsdale. We had thought that this connexion of lateral curvature with debility or quasi-paralysis of groups of respiratory muscles must be universally known by this time, and that the primary seat of the affection ought to be sought in the nervous centres, and especially in that portion from which the external respiratory nerves arise. Mr. Lonsdale will do well to read our review of Stromeyer's work on Paralysis of the Muscles of Inspiration (Vol. V, p. 133), or, better still, the original publication.

The mechanical support Mr. Lonsdale recommends is a modification of one invented by Mr. Tamplin. The pelvis (as in all these instruments) is the base of support; a crutch, arising from an apparatus attached to the pelvis (a "pelvis hook"), supports the left shoulder, and a pad attached to a lever taking its point of support from the same apparatus, is compressed by means of a screw against the bulging ribs of the right side. No numerical details inform us as to the success with which this machine has been used, but it is probably neither greater nor less than that of others of the same kind. It looks ingenious on paper.

This machine is the main agent in the treatment; two other proceedings are adjuvant to it. First, a couch is devised, by which the patient is recumbent on her right side, the "bulging ribs" being compressed by means of a strap. This seems to be little more than a modification of the hammock recommended by Pravatz, of Paris, with indications precisely similar to those stated by Mr. Lonsdale. Secondly, a plan is recommended whereby the muscles of the back are brought into free action. Mr. Lonsdale observes, that in the confirmed cases of scoliosis exercise is injurious.

There is not a word in the book as to the constitutional treatment of the affection. We are inclined to think that Mr. Lonsdale can produce a better book than this; but he must eschew mere mechanics, and study the living machinery of respiration in all its relations. Of the mode he has partly an example in Stromeyer's works; without this, no work on deformities can rise above the barest mediocrity.

---

